# Supplementary material for: From What to How: An Initial Review of Publicly Available AI Ethics Tools, Methods and Research to Translate Principles into Practices
Source: Sci Eng Ethics. 2019 Dec 11;26(4):2141–68. doi: 10.1007/s11948-019-00165-5 (PMC7417387; doi:10.1007/s11948-019-00165-5)
Supplement: Supplementary file 1 — Supplementary material 1 (DOCX 83 kb) [file 11948_2019_165_MOESM1_ESM.docx]

| **Typology** | | | | | | | |
| --- | --- | --- | --- | --- | --- | --- | --- |
|  | **Business and use-case development**  Problem/improvements are defined and use of AI is proposed | **Design Phase**  The business case is turned into design requirements for engineers | **Training and test data procurement**  Initial data sets are obtained to train and test the model | **Building**  AI application is built | **Testing**  The system is tested | **Deployment**  When the AI system goes live | **Monitoring**  Performance of the system is assessed |
| **Beneficence**  Stakeholder participation:  Protection of fundamental rights  Sustainable and environmentally friendly AI  Justification**:** | **[27] [37] [41] [46] [47] [64] [80] [84] [90] [94]** Conditions 8 and 9 of overarching framework. **[98] [104]** | **[16] [34] [45] [66]** | **[3] [31]** | **[9]** | **[50]** |  | **[10]** |
| **Non-Maleficence**  Resilience to attack and security  Fallback plan and general safety  Accuracy  Privacy and Data Protection:  Reliability and Reproducibility  Quality and integrity of the data  Social Impact | **[6] [19] [94]** Conditions 1-7 of overall framework **[105]** | **[1] [67] [91]** | **[7] [40] [49] [68] [73] [92] [106]** | **[12] [51] [53] [69] [82]** | **[23] [93]** | **[4] [5] [56] [76]** | **[60] [65]** |
| **Autonomy**  **Human agency**  **Human oversight** | **[94]** conditions 10 through 15 of the overall framework | **[17] [44] [74] [75]** |  |  |  | **[17] [70] [96]** |  |
| **Justice**  Avoidance of unfair bias  Accessibility and universal design  Society and democracy  Auditability  Minimisation and reporting of negative impacts  Trade-offs  Redress | **[14] [62] [25] [94]** Conditions 12 through 16 of the overall framework. |  | **[13] [15] [42] [43] [44] [95]** | **[2] [18] [33] [57] [59] [95] [102]** | **[20] [31] [32] [55] [71] [81] [95] [97] [99]** | **[21] [53]** | **[24] [26] [29] [85] [89]** |
| **Explicability**    Traceability  Explainability  Interpretability | **[25]** | **[53] [93]**  . |  | **[37] [39] [54] [101]** | **[11] [22] [38] [39] [58] [61] [72] [79] [86] [87] [88] [101] [103]** | **[4] [8] [35] [63] [77]** | **[25] [52] [96] [101]** |

| **Index** | |
| --- | --- |
| **Tool** | **Description** |
| 1. a3i the Trust in AI Framework | The trust in AI framework is based on three components: Data, infrastructure, model and helps organisations to design their systems to be safe, secure and explainable by building these elements into each of the three components. |
| 1. Agarwal et al (2018) A Reductions approach to fair classification | A general-purpose methodology for approaching fairness. Using binary classification, the method applies constraints to reduce fair classification to a sequence of cost-sensitive classification problems. Whose solutions yield a randomized classifier with the lowest (empirical) error subject to the desired constraints.  A python implementation can be found here: <https://github.com/Microsoft/fairlearn> |
| 1. AI Commons | AI Commons is a non-profit organisation which aims to bring together problem owners and the community of AI within a ‘safe sandbox’ environment for collaboration and solution evaluation. In addition, the research labs can provide trust data repositories and access to cloud and compute capabilities.  More information can be found here: <https://aicommons.com/> |
| 1. AI Now Institute Algorithmic Accountability Policy Toolkit | The Toolkit is designed to provide legal and policy advocates with a basic understanding of government use of algorithms. In addition the toolkit contains resources for advocates interested in or currently engaged in work to uncover where algorithms are being used and to create transparency and accountability mechanisms.  The toolkit is available here: <https://ainowinstitute.org/aap-toolkit.pdf> |
| 1. AI-RFX Procurement Framework | The Procurement framework is a set of templates that were put together by domain experts to support industry practitioners looking to procure AI systems in a way that ensures systems procured meet the bar for AI safety, quality and performance. It’s based on the Principles for Responsible Machine Learning (which the frameworks use as a list). The 8 principles are:   1. Human augmentation 2. Bias evaluation 3. Explainability by justification 4. Reproducible operations 5. Displacement strategy 6. Practical accuracy 7. Trust by privacy 8. Data risk awareness   The framework can be accessed here: <https://ethical.institute/rfx.html#overview> |
| 1. Alshammari and Simpson (2017) Towards a principled approach for engineering by design | In this paper, the authors analyse 3 privacy-related engineering requirements and convert them into a set of criteria that help developers identify data-processing activities that may lead to privacy violations and harms and in this way act as a means of specifying appropriate design decisions. Building from this, the authors also outline a preliminary principled framework for privacy by design |
| 1. Antignac et al (2016) Data Minimisation: a Language-Based Approach | In this paper, the authors present different mechanisms and architectures to ensure data minimisation and show how it is possible to compare minimisers so that developers can identify the best one for their specific task. |
| 1. Arnold et al (2018) FactSheets: Increasing Trust in AI Services through supplier’s declarations of conformity | The FactSheet concept is based on the use of supplier’s declarations of conformity in other industries. The FactSheet would contain a comprehensive set of declarations that demonstrate to consumers whether suppliers have met specific requirements. The items in the FactSheet are grouped into several categories aligned with the elements of trust:   1. Statement of purpose 2. Basic purpose 3. Safety 4. Security 5. Lineage   There are worked examples given in the paper. |
| 1. Arnold et al (2017a) Value alignment or misalignment – what will keep systems accountable? | In this paper the authors set out a hybrid approach for computational architectures, involving explicit storage of and reasoning abut moral norms and injunctions (written in the language of logic) to enable reinforcement learning algorithms to ‘learn’ how to make norm-aligned decisions on a case by case basis. |
| 1. Arnold et al (2018b) The “big red button” is too late: an alternative model for the ethical evaluation of AI systems | In this paper the authors set out a computational method for providing algorithmic systems with an ‘ethical core’ that would continually test the system, without the system knowing, to check that it was making decisions in an ethically-optimised manner in a range of different scenarios. |
| 1. Bach et al (2015) On Pixel-Wise Explanations for Non-Linear Classifier Decisions by layer-wise relevance propagation | This paper presents an approach to explainability, outlining a methodology that allows developers to visualise the contributions in individual image pixels to the overall prediction made by an image classifier. |
| 1. Bassily et al (2018) Model-Agnostic Private Learning via stability | This paper sets out an experimental method, based on differential privacy, that enables a classifier to answer as many classification queries as possible whilst ensuring the privacy of the data set. The approach is intended to enable the creation of privatized predictions with minimal negative impact on accuracy. |
| 1. Bender and Friedman (2018) Data statements for natural language processing: toward migrating system bias and enabling better science | The authors of this paper suggest that developers use a form known as a data statement to make decisions about: curation, language variety, text characteristics etc. of a data set used in the training of an NLP algorithm. Although presented as being NLP specific this method could be applied more broadly. |
| 1. Binns (2018) Algorithmic Accountability and Public Reason | Binns argues that the concept of ‘public reason’ could be used as a constraint on algorithmic decision-making, suggesting that developers ensure that an algorithmic system’s outputs must be transparent and must be based n epistemic and normative standards that would be acceptable to all reasonable people. The paper argues that by taking this approach, developers could help ensure that algorithmic decisions are made on common principles. |
| 1. Bolukbasi et al (2016) Man is to computer programmer as woman is to homemaker? | This paper outlines a methodology for modifying word embeddings to remove gender stereotypes. An implementation of the methodology can be accessed here: <https://github.com/tolga-b/debiaswe> |
| 1. Butnaru et al (2018) Humans in AI Trello Board | Set up like a Trello Board, Butnaru and colleagues have developed an 8-step process to assess whether a problem is totable for an ML solution and how to design from the beginning when and where ethical considerations will need to be thought about and by whom. It is based on the principles provided in the IEEE’s ethically aligned design.  The Trello Board is accessible here: <https://humansinai.com/>  <https://trello.com/b/SarLFYOd/agile-ethics-for-ai-hai> |
| 1. Butterworth (2018) the ICO and artificial intelligence. The role of fairness in the GDPR framework | This paper outlines the five key areas of concern that the ICO’s guidance to AI fairness and the GDPR:   1. The use of algorithms to discover correlations rather than the traditional approach of seeking to validate correlations 2. The opacity of processing 3. The tendency to collect all the data 4. The repurposing of data 5. The use of new types of data   And highlights how developers should be away of purpose limitation, data minimisation and consent – stressing the technical challenges involved in this and what developers can do to tackle these. |
| 1. Calders and Verwer (2010). Three naïve Bayes approaches for discrimination-free classification | This paper presents three approaches for making naïve Bayes classifiers non-biased:   1. Modifying the probability of the decision being positive 2. Training one model for every sensitive attribute value and balancing them 3. Adding a latent variable to the Bayesian model that represents the unbiased label and optimising the model parameters for likelihood using expectation maximisation |
| 1. Cavoukin et al (2010) Privacy by design: essential for organisational accountability and strong business practices. | This paper outlines the principles of privacy by design practices and argues that these principles can be used as criteria for building privacy and accountability into organizational information management practices.  The principles are:   1. Proactive not reactive; preventative not reactive 2. Privacy as the default 3. Privacy embedded into design 4. Full functionality – positive sum, not zero-sum 5. End-to-end lifecycle protection 6. Visibility and transparency 7. Respect for user privacy |
| 1. Chowdhury (2019) Tackling the challenge of ethics in AI fairness Tool. (Accenture) | The Accenture AI Fairness Toolkit, developed with the ATI data study group, scrutinises data that goes into an algorithm to identify and remove any coordinated influence that may lead to an unfair outcome – rather than simply highlighting the problem, it aims to fix it.  Secondly, the toolkit analyses the model itself to check that false positives and false negatives are fairly distributed and suggests ‘fixes’ if it finds problems. |
| 1. Citron and Pasquale (2014) The Scored Society: Due process for automated predictions | In this paper, the authors set out procedural safeguards for automated scoring systems (e.g. credit scoring) based on transparency to facilitate testing, risk assessment and reports, interactive modelling and notice guaranteed by audit trails to individuals |
| 1. Datta et al (2017) Algorithmic transparency via quantitative input influence | This paper introduces an explainability or ‘transparency’ method known as Quantitative Input Influence (QII). This method uses a series of measures that capture the degree f influence of inputs on outputs, using principled aggregated measures e.g. the Shapley value and paying particular attention to correlated inputs. These measures are then used as the basis for transparency reporting. |
| 1. Dennis et al (2016) Practical verification of decision-making in agent-based autonomous systems | This method introduces a verification methodology, based on the use of program model checkers, to analyse the decision-making component in agent-based hybrid systems. |
| 1. Diakopoulos (2015) Algorithmic accountability: journalistic investigation of computation power structures | This study provides five examples of algorithmic accountability reporting involving the use of reverse engineering methods (i.e. the process of articulating the specifications of a system through a rigorous examination drawing n domain knowledge, observation, and deduction to unearth a model of how that system works) in journalism |
| 1. Diakopoulos et al. Principles for Accountable Algorithms and Statement for algorithms | In this web resource, the authors set out five guiding principles for the creation of accountable algorithms and then provide guiding questions to ask under each of these headings at design stage, pre-launch stage and post-launch stage. The intention is that the answers to these questions become a social impact statement for the algorithmic system that has been built.  Accessible from this link: <http://www.fatml.org/resources/principles-for-accountable-algorithms> |
| 1. Diakopoulos et al. Algorithm Tips - Resources and Leads for investigating algorithms in society | The Algorithm Tips website provides a wealth of resources, including a curated dataset of algorithms being used in the US Federal government, to help individuals investigate algorithm use for the purposes of transparency. There is also a supporting paper on how the database was curated.  The webpage is here: <http://algorithmtips.org/resources/> |
| 1. DotEveryone. The Consequence Scanning Event | Consequence scanning is an agile event (or agile ceremony) which is designed to help tech teams create products or services that are aligned with their organisation’s values and cultures. There is a manual on how to run the event, but the aim is to get teams (including teams of developers) to answer the following three questions:   1. What are the intended and unintended consequences of this product or feature? 2. What are the positive consequences we want to focus on? 3. What are the consequences we want to mitigate?   More details can be found in the manual which can be downloaded from: <https://doteveryone.org.uk/project/consequence-scanning/> |
| 1. Ellpha | Ellpha is a community of people campaigning for the creation of more gender-neutral algorithmic systems <https://www.ellpha.com/what-is-ellpha> |
| 1. Epstein (2018) TuringBox: An experimental Platform for the evaluation of AI systems | This paper introduces TuringBox as a means of auditing black-box AI systems. On one side of the platform, contributors upload algorithms in various forms. TuringBox automatically compares a newly uploaded algorithm to others on the platform in terms of accuracy, fairness or other mechanisms it deems appropriate. Contributors gain reputation points on the platform as a function of their algorithm’s performance in these categories. On the other side of the platform, examiners investigate the output of algorithms. |
| 1. Equity Evaluation Corpus | The Equity Evaluation Corpus (EEC) is a database consisting of 8,640 sentences carefully chosen to highlight biases towards specific races and/or genders and is used to examine automatic sentiment analysis systems.  More information is available here: <https://saifmohammad.com/WebPages/Biases-SA.html> |
| 1. Ethics Net | Ethics Net is a non-profit organisation that is aiming to construct, collate and annotate a range of datasets that reflect many different cultures, opinions and creeds, which can expand in scope and nuance over time. The aim is to teach prosocial behaviours and preferences to machines. The first prototype is based on ethical deliberation and entitled ‘GENETH’ based on a paper by Anderson and Anderson.  More detail available on this link: <https://www.ethicsnet.com/about> |
| 1. Feldman et al (2014) Certifying and removing disparate impact | This paper covers a broad range of topics but for the typology, it makes two important contributions:   1. It introduces a test for disparate impact based on how well a protected class can be predicted from other attributes 2. It describes a variety of methods that can be used to make data unbiased |
| 1. Fish et al (2016) A confidence-based approach for balancing fairness and accuracy | This paper outlines research which aims to improve the fairness of adaptive boosting, support vector machines and logistic regression ML algorithms without compromising their accuracy. It makes two contributions:  The first is a method for achieving fairness by shifting the decision boundary for the protected group.  The second is the introduction of a new measure of fairness ‘resilience to random bias) which the authors claim provides a more complete picture of the fairness of an algorithm than other existing fairness measures. |
| 1. Friedman et al (2017) a survey of value sensitive design methods | This paper defines value sensitive design as ‘a theoretically grounded approach to the design of technology that accounts for human values in a principled and systematic manner throughout the design process. It details 14 different specific methods:   1. Direct and indirect stakeholder analysis 2. Value source analysis 3. Co-evolution of technology and social structure 4. Value scenario 5. Value sketch 6. Value-oriented semi-structured interview 7. Scalable information dimensions 8. Value-oriented coding manual 9. Value-oriented mock-up, prototype or field deployment 10. Ethnographically informed inquiry regarding values and technology 11. Model of informed consent outline 12. Value dams and flows 13. Value sensitive action-reflection model 14. Envisioning cards |
| 1. Gebru (2018) Datasheets for Datasets | This paper recommends that every dataset used to train an ML model be accompanied with a datasheet documenting its creation, composition, intended uses, maintenance and other properties. The paper provides several examples of what this would look like. |
| 1. Glenn. Futures Wheel | The Futures Wheel is a technique, originally developed in 1971 and now included within the ‘Ethics Kit’ , to help developers visualise the direct and indirect consequences of a particular development.  It can be found in the Ethics Kit here: <http://ethicskit.org/futures-wheel.html> |
| 1. Goldstein et al (2014) Peeking inside the black box: visualising statistical learning with plots of individual conditional expectation | This paper outlines the methodology for creating Individual Conditional Expectation (ICE) plots as a means of visualising the model estimated by any ML algorithm. The paper includes several worked examples to demonstrate how the methodology can be used in practice. |
| 1. Guidotti et al (2018) A survey of methods for explaining black box models | This paper sets out the desiderata of an interpretable model. An interpretable model must take into account:   - A mechanism for measuring the interpretability of model – the degree to which it can be understood by humans - A measure for the accuracy of the model - A measure of comparison against black-box version of the algorithm   The paper then outlines a variety of the techniques available for enhancing interpretability:   - Decision tree or single tree - Decision rules - Features importance - Saliency map - Sensitivity analysis - Partial dependence plot - Prototype selection - Activation maximation |
| 1. Hall and Gill. H20.ai Machine Learning Interpretability Resources | This is an online toolkit providing a range of resources (e.g. codebooks) available for use for the purpose of improving the interpretability of a an algorithm. have created a series of Juptyer notebooks using open source tools including Python, H20, XGBoost, GraphViz, Pandas, and NumPy to outline practical explanatory techniques for machine learning models and results. The notebooks cover the following techniques:  -Monotonically constrained GBM,s partial dependence, and ICE: which all use monotonicity constraints to train an explainable, and potentially regulator-approvable GVM model, by ensuring predictions only increase or only decrease for any change in a given input variable.  <https://github.com/h2oai/mli-resources/blob/master/notebooks/mono_xgboost.ipynb>  <https://github.com/h2oai/mli-resources/blob/master/notebooks/pdp_ice.ipynb> |
| 1. Hazy | Hazy is a start-up that creates synthetic datasets off the data provided to them by another company to enable the protection of the initial dataset  More information can be found on their website: <https://hazy.com/> |
| 1. Hesketh. Ethics Cards | Ethics Cards is a car sorting tool to help developers think through ethical considerations that might arise during a sensitive project. The cards cover considerations for:   - Project kick-off - Finding people for research - Managing data and generating informed consent   The cards can be accessed in the ethics kit here: <http://ethicskit.org/downloads/ethicskit-cards.pdf> |
| 1. Holland et al (2018). The Dataset Nutrition Label: A framework to drive higher data quality standards | Similar to a nutrition label on food, the dataset nutrition label framework aims to create a standard methodology for highlighting the key ingredients of a dataset (E.g. meta-data, populations’, missing data, comparisons to other ‘ground-truth’ datasets) before it is used to train an AI model. The paper describes in detail the process of creating 7 prototypes.  A live version of the prototype can be accessed on this link:  <https://ahmedhosny.github.io/datanutrition/> |
| 1. ICOa Anonymisation: managing data protection risk. Code of Practice | This code of practice explains the issues surrounding the anonymisation of personal data, and the disclosure of data once it has been anonymised. It explains the relevant legal concepts and tests and provides good practice advice that is relevant to all organisations (and individuals) that need to convert personal data into a form in which individuals are no longer identifiable.  The guide can be accessed here: <https://ico.org.uk/media/1061/anonymisation-code.pdf> |
| 1. ICOb Guide to the General Data Protection Regulation (GDPR) | The Guide to the GDPR explains the provisions of the GDPR in detail to help organisations (and in the context of the typology, developers) comply with its requirements. Embedded within it is a data protection self-assessment toolkit.  The guide can be accessed here: <https://ico.org.uk/for-organisations/guide-to-data-protection/guide-to-the-general-data-protection-regulation-gdpr/> |
| 1. Ideo.org. The Field Guide to Human-Centred Design | The Field guide provides a range of tools to help developers design from a ‘human-centric’ perspective i.e. developing with communities so that they understand the people they are trying to serve and creating innovative solutions that people actually need. Tools include:   - The Frame your design worksheet - Guide to User research interviews - Card sort methodology for identifying what’s most important to the people they’re designing for   The Design Guide can be accessed from this link: <http://www.designkit.org/resources/1> |
| 1. IEEE (2019) Ethically Aligned Design | The aim of the IEEE Global Initiative on Ethics of Autonomous and Intelligent Systems is that ethically aligned design will provide pragmatic and directional insights and recommendations, serving as a key reference for the work of developers, educators and policymakers.  The General Principles of Ethically Aligned Design are:   1. Human rights 2. Well-being 3. Data agency 4. Effectiveness 5. Transparency 6. Accountability 7. Awareness of Misuse 8. Competence   These principles are mapped to a series of practical and, in some cases, technical recommendations designed to move the conversation from principles to practice.  There is also an entire section in Ethically Aligned Design covering methods to guide ethical research and design. These are primarily designed to be used by organisations but developers may find them useful as overall guides. |
| 1. Involve & DeepMind, How to stimulate effective public engagement on the ethics of AI | This report is the output of a research project led by Involve and DeepMind to explore how more sustained public engagement on the ethics of the use of AI in public service delivery might be stimulated. It brings together participant recommendations on the themes of:   - Framing the debate - Governance and decision-making - Communications and language - Practical application of engagement - Moving the agenda forward - Reflections and learning |
| 1. Johansson et al (2016) Learning Representation for Counterfactual Inference | This paper outlines a methodology for creating a causal framework that can be used to model the relationship between protected attributes and data to test how well a model addresses causality in fairness. |
| 1. Joshi et al (2019) Generative Adversarial Networks (GANs) for synthetic dataset generation with binary classes | The research outlined in this paper focuses on the idea of using generative adversarial networks (GANs) to generate synthetic substitute datasets that closely resemble the real data in situations where data is limited or in situations where it is desirable to minimise access to the real data |
| 1. Kleinberg et al (2017). Human decisions and Machine Predictions | The authors stress that in situations where an ML solution is deployed, it is also important to consider the relationship between prediction and decision, as well as the relationship between data and prediction as this can have an equally large effect on the impact (positive or negative) of the deployed system. |
| 1. Kolter and Madry. Materials for tutorial Adversarial Robustness: Theory and Practice | This is an online tutorial comprised of a range of resources (including a video) that help developers develop classifiers that are robust to real-time perturbations of their inputs.  The tutorial can be accessed here: <https://adversarial-ml-tutorial.org/> |
| 1. Kroll (2018) The Fallacy of Inscrutability | The author of this paper argues that the creation of inscrutable black-box algorithms is a design decision that can be effectively avoided and is, therefore, akin to malpractice. Kroll argues that a system’s inputs, outputs and outcomes should be regularly reviewed and audited to uncover bugs, biases and incorrect assumptions (i.e. problems with construct validity) as well as checking for concept drift. |
| 1. Kroll et al (2017) Accountable Algorithms | This paper provides a very detailed overview of the approaches available to developers to document their code in a way that makes it easier to analyse, scrutinise and correct. It then provides further detail on methods available to developers for evaluating systems – both static and dynamic methods. Finally the paper descirbes a series of technical tools that can be used buy developers to help ensure that their system meets the requirement of ‘procedural regulatory’ i.e. it constantly makes decisions based on the same rule. This framework is based on:   1. Software verification 2. Cryptographic commitments 3. Zero-knowledge proofs 4. Fair random choices |
| 1. Kusner et al (2017) Counterfactual Fairness | This paper is another exploration of how to use causal inference techniques to design algorithms that accurately address the underlying cause of issues with fairness |
| 1. Lakkaraju et al (2017) The Selective Labels Problem: Evaluating Algorithmic Predictions in the Presence of Unobservable | This paper develops a novel technique called contraction to act as an evaluation metric, failure rate vs. acceptance rate, that can be used to effectively compare human decisions with algorithmic predictions even when not all the elements involved in the algorithmic prediction are known. |
| 1. Lepri et al (2018) Fair, Transparent, and Accountable Algorithmic Decision-making Processes: the premise, the proposed solutions and the open challenges. | This paper provides a detailed overview of (largely) statistical techniques available to developers to prevent algorithmic discrimination and maximise fairness and those designed to help improve transparency and accountability. It then describes, in detail, the Open Algorithms (OPAL) project.  OPAL is a socio-technological platform led by several partners that aims to leverage private sector data for public good purposes by providing a mechanism for public sector organisations to run queries (algorithms) on privately held datasets without ever revealing the underlying dataset, and brining the two organisations together to co-design the solution.  More information about the OPAL project can be found here: <https://www.opalproject.org/> |
| 1. Li et al (2017) Deep learning for case-based reasoning through prototypes: a neural network that explains its predictions | This paper outlines a specific network architecture for deep learning that the authors claim naturally explains its own reasoning for each prediction. |
| 1. Lundberg and Lee (2017) A unified approach to interpreting model predictions | The authors outline a unified approach to model explainability known as SHAP (shapely regression values and layer-wise relevance propagation) which combines the methodologies of local-interpretable model explainers, DeepLIFT (<https://github.com/kundajelab/deeplift>), Tree Interpreters, QII and shapely sampling values to deliver a method that they clam can be used to explain the prediction of any machine learning model.  The library is available here: <https://github.com/slundberg/shap> |
| 1. Madras et al (2018) learning adversarially fair and transferable representations | The authors outline a method for representation learning, using adversaries, to ‘teach’ an algorithmic system to learn how to be fair in all situations, even when the predictor is run on unseen tasks. |
| 1. Makri and Lambrinoudakis (2015) Privacy Principles: Towards a common privacy audit methodology | This paper attempts to create a standardised, repeatable method for privacy audits by collecting and classifying all known privacy principles and requirements and identifying the way each privacy requirement can be satisfied and he sequence with which each privacy requirement should be addressed based on a prioritisation. I outlines that the most common and widely accepted privacy principles are used in the structured privacy audit:     - Purpose specification - Collection limitation - Data quality - Use, retention and disclosure limitation - Security safeguards - Openness - Individual participation - Accountability |
| 1. Microsoft – InterpretML | InterpretML is an open-sourced code toolkit (available on GitHub) aimed at improving explainability. |
| 1. MIT Moral Machines | MIT’s moral machine is a platform for 1) building a crowd-sourced picture of human opinion on how machines should make decisions when faced with moral dilemmas, and 2) crowd-sourcing assembly and discussion of potential scenarios of moral consequence. |
| 1. Mitchell et al (2019). Model Cards for Model Reporting | Similar and complementary to, ‘Datasets for Datasheets’, this paper introduces the concept of model cards – records that are one-to-two pages in length detailing trained model characteristics such as the type of model, intended use cases, information about attributes fr which model performance may vary, and measures of model performance. Suggestion sections of the model cards are included:   - Model details - Intended use - Factors - Metrics - Evaluation data - Training data - Quantitative analyses - Ethical consideration - Caveats and recommendations |
| 1. New Economic Impact Model | The aim of the New Economic Impact Model is to help developers assess the potential of proposed tech products to displace human rights and universal needs.  It can be found in the Ethics Kit here: <http://ethicskit.org/downloads/economy-impact-model.pdf> |
| 1. Nicolae et al (2018). Adversarial robustness toolkit | The Adversarial Robustness Toolbox (ART) is a python library designed to support researchers and developers in creating novel defence techniques, as well as in deploying practical defences of real-world AI systems. It is primarily focused on improving the adversarial robustness of visual recognition systems – but there are plans to further develop it.  The toolkit is accessible here: <https://github.com/IBM/adversarial-robustness-toolbox> |
| 1. ODI Data Ethics Canvas | The Data Ethics Canvas is designed to be used by anyone who collects, shares, and uses data (including developers) to help them identify and manage potential ethical issues – particularly at the start of a project. It is comprised of 15 sections that can be looked at in any order and prompts developers to:   - Describe the data project, model or practice and its purpose - Identify people likely to be affected by the activities - Assess potential risks and limitations associated with the activities, which could negatively affect people and society - Plan steps that will need to be taken to mitigate those risks and limitations   Further detail (including the user manual) can be found via these links: <http://tinyurl.com/DECanvas>  <http://tinyurl.com/DEUserGuide> |
| 1. Oetzel and Speikermann (2014) A systematic methodology for privacy impact assessments: a design science based approach | This paper lays out a step-by-step privacy impact assessment (PIA) to help developers achieve ‘privacy-by-design.’ The assessment is made up of 7 stages – each of which is detailed more fully in the paper:   1. Characterisation of the system 2. Definition of privacy targets 3. Evaluation of degree of protection demand for each privacy target 4. Identification of threats for each privacy target 5. Identification and recommendation of existing or new controls suited to protect against threats 6. Assessment and Documentation of residual risks 7. Documentation of PIA process |
| 1. ONS. The ONS Methodology working paper on synthetic data | This paper highlights tools that can be used to produce synthetic data for specific user requirements.  More information via this link:  <https://www.ons.gov.uk/methodology/methodologicalpublications/generalmethodology/onsworkingpaperseries/onsmethodologyworkingpaperseriesnumber16syntheticdatapilot> |
| 1. OpenMined | OpenMined is an open source community, with a well-documented repository on GitHub, that contributes to code-based solutions to aspects related to algorithmic safety – in particular privacy protection. The community are creating an accessible ecosystem of tools for private, secure, multi-owner governed AI by extending popular libraries like TensorFlow and PyTorch with advanced techniques in cryptography and private machine learning including: federated learning, differential privacy, multi-party computation, homomorphic encryption, consensus and threshold governance.  <https://www.openmined.org/>  <https://github.com/OpenMined> |
| 1. Orcutt (2017) Personal AI Privacy Watchdog could help you regain control of your data | As described by Orcutt in this blog, privacy assistant is the result of a research project by Carnegie Mellon University. Users answer questions e.g. “in general, do you feel comfortable with finance apps accessing your location” and analyses the user’s apps on their phone. It then uses ML to make specific recommendations about how the user should manage their permissions.  The blog can be accessed here: <https://www.technologyreview.com/s/607830/personal-ai-privacy-watchdog-could-help-you-regain-control-of-your-data/> |
| 1. Overdorf et al (2018) Questioning the assumptions behind fairness | In this paper, the authors outline protective optimisation techniques that provide explicit modelling and evaluation of the impact of optimisation systems on populations and environments. These methods are designed to protect against attacks on fairness from adversarial external enemies e.g. attempts to ‘game the system’ |
| 1. Oxborugh et al. Explainable AI: Driving business value through greater understanding | This PWC report provides a high-level introduction to the range of techniques available to developers seeking to make their models more explainable. It splits the methods that it covers thus:  Model-agnostic approaches:   - Sensitivity analysis - LIME (see <https://github.com/h2oai/mli-resources/blob/master/notebooks/lime.ipynb>) - SHAP   Algorithm-specific methods:   - TreeInterpreters (see <https://github.com/andosa/treeinterpreter> ; <https://github.com/TeamHG-Memex/eli5> - Neural Network Interpreters - Activation Maximation   Where there are available open-source implementations of these techniques, these are highlighted in the typology |
| 1. Papernot et al (2018) Scalable Private Learning with PATE | PATE is an acronym for Private Aggregation of Teacher Ensembles. The paper discusses how in the PATE approach multiple teachers are trained on disjoint sensitive data (e.g. different user’s data) and uses the teachers’ aggregate consensus answers in a black-box fashion to supervise the training of a ‘student’ model. Although this is an existing research paradigm, the authors in this paper show how to scale the approach. |
| 1. Peters and Calvo (2019) Beyond principles: a process for responsible tech. | The authors introduce ‘R2D2’ - the responsible rendition of the double diamond design framework. Rather than attempting to dictate very specific approaches to design, it provides a loose framework for when specific issues should be considered during the design process and suggests some tools that are available to help with this.  It can be accessed via this link: <https://tinyurl.com/y553peo5> |
| 1. Peters et al (2018). Designing for motivation, engagement, and wellbeing in digital experience | In this web article, the authors argue that in order for ML solutions to protect (and hopefully improve) a user’s wellbeing, they must ensure that they consider how the ML solution impacts at five different levels:   1. At the point of adoption 2. At the point of interaction with the interface 3. When the user is engaging with the platform to complete specific tasks 4. As part of the technology-supported behaviour 5. As part of the user’s life overall   These five levels (or spheres as they are called in the paper) sit within society which is the 6^th^ level of analysis.  The article and an illustrative diagram can be accessed here: <https://www.frontiersin.org/files/Articles/300159/fpsyg-09-00797-HTML/image_m/fpsyg-09-00797-t001.jpg> |
| 1. Pineau (2019) The Machine Learning Reproducibility Checklist | This is a very simple checklist which helps prompts developers to take steps in the design of their model to ensure that it is reproducible.  It is available here: <https://www.cs.mcgill.ca/~jpineau/ReproducibilityChecklist.pdf> |
| 1. Reisman et al (2018) Algorithmic Impact Assessments: A practical Framework for Public Agency Accountability | The authors introduce algorithmic impact assessments that are designed to be used by public agencies (but can be used by developers to understand what the ‘bar’ is) to give them more insight int the systems that they are procuring and help ensure the public are involved transparently throughout so that the public can hold both the developers and the procurers accountable. |
| 1. Responsible AI Licenses | The Responsible AI Licenses work in the same way as end-user license agreements and are designed to provide developers with a mechanism for restricting the use of their AI solution to prevent it being used for irresponsible or harmful purposes. Two different licenses are available:   - - Source code license:     - Allows developers to gain most of the benefits of open-source while mitigating the risk of releasing powerful code into the wild for anyone to use   - End-user license:     - Governs how customers use a software package.   The Licenses can be accessed here: <https://www.licenses.ai/ai-licenses> |
| 1. Ribeiro (2016) ‘Why should I trust you?’ Explaining the predictions of any classifier | In this paper, the authors introduce the model explanation method – LIME – describe its methodology, and how it can be used to provide an explanation for the predictions of *any* classifier, by learning an interpretable model locally around the prediction. It also describes a method for explaining models by framing the task of providing an explanation as a submodular optimisation problem.  The Python Library is available here:  <https://github.com/marcotcr/lime> |
| 1. Royal Society and British Academy. Data Management and use: Governance in the 21^st^ Century | This report sets out a data management and data use governance framework grouped around three categories of functions:   1. Anticipate, monitor and evaluate 2. Build practices and set standards 3. Clarify, enforce and remedy |
| 1. Russel et al (2017) When Worlds Collide: Integrating Different Counterfactual Assumptions in Fairness | This paper uses the broad concept of counterfactual fairness to develop a methodology that frames the need to train a fair learning classifier, as an optimisation problem with fairness constraints. |
| 1. Ryffel et al (2018) A generic framework for privacy preserving deep leaning | This paper introduces a new framework/architecture for privacy preserving deep learning by discussing how developers can implement constructs such as:   - Federated Learning - Secure Multiparty Computation - Differential Privacy |
| 1. Saleiro et al (2018). Aequitas: A bias and Fairness Audit Toolkit | Aequitas is an open source bias and fairness audit toolkit that was released in 2018. It is designed to enable developers to seamlessly test models for a series of bias and fairness metrics in relation to multiple population sub-groups.  The toolkit can be accessed here: <https://github.com/dssg/aequitas> |
| 1. Sampson and Chapman (2019). AI needs an Ethical Compass | This blog from ideo.org sets out four design principles and ten activities designed to help guide am ethically responsible, culturally considerate, and humanistic approach to designing with data.  The principles are:   1. Data is not truth 2. Don’t presume the desirability of AI 3. Respect privacy and the collective good 4. Unintended consequences of AI are opportunities for design.   A series of cards matching the tasks to the principles are available for download from the link in the blog: <https://www.ideo.com/blog/ai-needs-an-ethical-compass-this-tool-can-help> |
| 1. Sandvig et al (2014) Auditing Algorithms: Research Methods for Detecting Discrimination on Internet Platforms | The authors in this paper outlines how to perform a range of different types of algorithmic audits:   - Code audit - Non-invasive user audit - Scraping audit - Sock Puppet audit - Crowdsourced audit/collaborative audit |
| 1. Seldon.io. Alibi release | Alibi is an open source Python library aimed at ML model inspection and interpretation. It focuses on providing the code needed to produce explanations for black-box algorithms. The goals of the library are to:   - Provide high quality reference implementations of black-box ML model explanation algorithms - Define a consistent API for interpretable ML models - Support multiple use cases (e.g. tabular, text and image data classification, regression) - Implement the latest model explanation, concept drift algorithmic bias detection and other ML model monitoring and interpretation methods   The Library can be accessed here: <https://github.com/SeldonIO/alibi>  . |
| 1. Shrikumar et al (2017). Learning important features through propagating activation differences | This paper introduces DeepLIFT (Deep Learning Important FeaTures) as a method for ‘explaining’ the predictions made by neural networks.  The code is available via this link: <https://github.com/kundajelab/deeplift>  The video tutorial is available here: <https://www.youtube.com/playlist?list=PLJLjQOkqSRTP3cLB2cOOi_bQFw6KPGKML> |
| 1. Simonyan et al (2013) Deep Inside Convolutional Networks: Visualising image classification models and saliency maps | This paper provides a worked example and more detail on how to use the saliency mapping technique for explainability purposes |
| 1. Sokol and Flach (2018). Glass-box: Explaining AI decisions with counterfactual statements through conversations with a voice-enabled virtual assistant | This paper discusses the development and testing of a prototype voice-enabled device, called Glass-box which uses can question to get counterfactual answers to questions about how an automated decision was made. It is designed to be easy for lay members of the public to understand. |
| 1. Stahl and Wright (2018) Ethics and Privacy in AI and Big Data: Implementing Responsible Research and Innovation | The authors argue that the Responsible Research and Innovation processes can be used to help ensure various viewpoints are captured in the design of algorithmic systems. RRI is an approach to research and innovation governance that aims to ensure all outputs of the research are acceptable, desirable and sustainable. There is an open source and searchable RRI toolkit available from: <https://www.rri-tools.eu/search-engine> |
| 1. Suphakul and Senivongse (2017) Development of privacy design patterns based on privacy principles and UML | This paper takes the privacy by design concepts and turns them into easily implementable design patterns. |
| 1. TensorFlow Privacy | TensorFlow Privacy is a GitHub Library that is designed to make it easier for developers to train machine-learning models with privacy, and for researchers to advance the state of the art in MK with strong privacy guarantees. It is based on the principles of differentiated privacy. A technical whitepaper is available which describes the privacy mechanisms in more detail.  The Library can be accessed here: <https://github.com/tensorflow/privacy> |
| 1. The Turing Way | The Turing Way is an online open-sourced guide to reproducible data science. More information, including very detailed documentation, can be found here: <https://github.com/alan-turing-institute/the-turing-way> |
| 1. Van der Poel (2016) An ethical framework for evaluating experimental technology | The argument this paper makes, is that the introduction of technological systems (including ML systems) is akin to a social experiment. It provides an ethical framework for the acceptability of such experiments based on the bioethical principles of non-maleficence, beneficence, respect for autonomy, and justice. The framework is made up of 16 principles and is intended to act as a checklist for those regulating or overseeing these systems. It can be used by developers as a design pattern. |
| 1. Varshney (2018). IBM 360 Fairness | IBM 360 degree toolkit contains a comprehensive set of fairness metrics for datasets and machine learning models, explanations for these metrics, and algorithms to mitigate bias in datasets at the pre-processing and model training stages.  Extensive user guidance is available and detailed tutorials are provided:  <https://www.ibm.com/blogs/research/2018/09/ai-fairness-360/>  <https://github.com/IBM/aif360>  <http://aif360.mybluemix.net/resources> |
| 1. Wachter and Mittelstadt (2019). A right to reasonable inferences: re-thinking data protection law in the age of big data and AI | In this paper, the authors argue that it’s not enough just to protect the privacy of the literal data, but also the information that can be inferred about an individual as it is these inferences that can be especially harmful. |
| 1. Wachter et al (2017) Counterfactual explanations without opening the black box: Automated decisions and the GDPR | This paper describes the detailed statistical methodology for acting on the otherwise largely theoretical concept of counterfactuals. |
| 1. Wellcome Data Labs agile methodology | The Wellcome Data Labs agile methodology recommends embedding a social scientist within a multidisciplinary team of software developers who is given the explicit responsibility for testing algorithmic models for social impact |
| 1. Wexler (2018). The What-if-Tool: Code-Free Probing of Machine Learning | The What-if Tool from Google is an open-source TensorBoard web application which lets users analyse an ML model without writing code. It visualises counterfactuals so that users can compare a data-point to the most similar point where the model predicts a different result. In addition, users can explore the effects of different classification thresholds, taking into account constraints such as different numerical fairness criteria. There are a number of demos available – showing how the different functions work on pre-trained models.  <https://ai.googleblog.com/2018/09/the-what-if-tool-code-free-probing-of.html>  <https://pair-code.github.io/what-if-tool/> |
| 1. Wilson (2018) Auditing Algorithms @ Northeastern | The Auditing Algorithms website has details of projects run to inspect ‘live’ algorithms including (where available) the open-source data and code from the research projects to enable people to repeat the projects. |
| 1. XAI Library | XAI is a Machine Learning library that is designed with AI explainability in mind. It contains a wide variety of tools that enable developers to analyse and evaluate their data and models. It is maintained by The Institute for Ethical AI & ML and was developed based on the 8 principles of responsible machine learning from the Institute:   1. Human augmentation 2. Bias evaluation 3. Explainability by justification 4. Reproducible operations 5. Displacement strategy 6. Practical accuracy 7. Trust by privacy 8. Data risk awareness   The Library is available here: https://github.com/EthicalML/xai |
| 1. Zafar et al (2015). Fairness Constraints: Mechanisms for Fair Classification | This paper is another example of how to use constrains as a mechanism for training ‘fair’ learning classifiers |
| 1. Zhang & Zhu (2018) Visual interpretability for deep learning: a survey | In this paper the authors provide an overview of techniques available for understanding neural-networks by covering the following:   - - The visualisation of CNN representations   - Methods for diagnosing representations of pre-trained CNNs   - Approaches for disentangling pre-trained CNN representations   - Learning of CNNs with disentangled representations   - Middle-to-end learning based on model interpretability   A python implementation is available here: <https://github.com/mbilalzafar/fair-classification> |
| 1. Zhao (2018) Improving Social Responsibility of Artificial Intelligence by Using ISO 26000 | In this paper Zhao highlights how the **ISO 26000 Framework for Social Responsibility** can be used as a means of defining the organisation’s responsibility for the impacts of its decisions an activities on society, including thinking from the beginning of design issues related to: organisational governance, labour practices, human rights, the environment, fair operating practices, consumer rights and community involvement. |
| 1. Zook et al (2017). Ten simple rules for responsible big data research | This paper provides a high-level and simple-to-follow checklist that aims to help developers think through how to minimise the harm of their solutions :   1. Acknowledge that data are people and can do harm 2. Recognise that privacy is more than a binary value 3. Guard against the reidentification of your data 4. Practice ethical data sharing 5. Consider the strengths and limitations of your data; big does not automatically mean better 6. Debate the tough, ethical choices 7. Develop a code of conduct for your organisation, research community, or industry 8. Design your data and systems for auditability 9. Engage with the broader consequences of data and analysis practices 10. Know when to break these rules |
| 1. Zyskind et al (2015). Enigma: Decentralised Computation Platform with Guaranteed Privacy | Enigma is a peer-to-peer network, enabling different parties to jointly store and run computations on data while keeping the data completely private. It is based on a highly optimized version of secure multi-party computation.  More information can be found here: <https://enigma.co/>  There will be other similar products/companies using a version of this methodology. It is included in the typology for illustrative purposes. |

Bibliography

For a very comprehensive guide to the terminology used in this typology please refer to the Ethically Aligned Design Glossary produced by the IEEE designed to give multidisciplinary teams a shared resource for reference to terms which may have meanings that are discipline specific: <https://standards.ieee.org/content/dam/ieee-standards/standards/web/documents/other/ead1e_glossary.pdf>

For an overview of where AI is being applied currently see <https://deepindex.org/> which keeps track of what AI can do and where it is being applied

To keep up-to-date with research on ‘responsible and ethical AI’ it is worth following the work of the following research groups:

AI Now <https://ainowinstitute.org/>

Alan Turing Institute Data Ethics Group <https://www.turing.ac.uk/news/alan-turing-institute-data-ethics-group>

Association for the Advancement of Artificial Intelligence <http://www.aaai.org/>

The Ethics and Governance of Artificial Intelligence Initiative <https://aiethicsinitiative.org/>

Microsoft: Artificial Intelligence <https://www.microsoft.com/en-us/research/research-area/artificial-intelligence/>

Center for Human-Compatible AI <https://humancompatible.ai/>

Future of Humanity Institute <https://www.fhi.ox.ac.uk/>

People + AI Research (PAIR) <https://ai.google/research/teams/brain/pair>

Digital Ethics Lab: Every Bit As Good <https://digitalethicslab.oii.ox.ac.uk/>

DARPA: Defense Advanced Research Projects Agency Explainable AI (XAI) project: <https://www.darpa.mil/program/explainable-artificial-intelligence>

Data & Society <https://datasociety.net/>

Data Transparency Lab: <https://datatransparencylab.org/about/>

AI for the Common Good <https://allenai.org/>

AI Ethics Lab <http://aiethicslab.com/>

Partnership on AI: About Us <https://www.partnershiponai.org/about/>

Auditing Algorithms. Adding Accountability to Automated Authority <http://auditingalgorithms.science/?page_id=89>

OpenAI <https://openai.com/about/#mission>

FATML. Fairness, Accountability and Transparency in Machine Learning <https://www.fatml.org/>

Integrate.ai. (2018 ). Responsible AI in Consumer Enterprise <https://www.integrate.ai/responsible-ai-in-consumer-enterprise>

Stanford, A. S. Center for AI Safety <http://aisafety.stanford.edu/>

RSA and DeepMind. Forum for Ethical AI <https://www.thersa.org/action-and-research/rsa-projects/economy-enterprise-manufacturing-folder/forum-for-ethical-AI>

a3i. (n.d.). The Trust-in-AI Framework. Retrieved from http://a3i.ai/trust-in-ai

Agarwal, A., Beygelzimer, A., Dudík, M., Langford, J., & Wallach, H. (2018). A Reductions Approach to Fair Classification. *ArXiv:1803.02453 [Cs]*. Retrieved from http://arxiv.org/abs/1803.02453

AI Commons. (n.d.). Retrieved from AI Commons website: https://aicommons.com/

*AI Now Institute Algorithmic Accountability Policy Toolkit*. (n.d.). Retrieved from https://ainowinstitute.org/aap-toolkit.pdf

AI-RFX Procuement Framework. (n.d.). Retrieved from https://ethical.institute/rfx.html

Alshammari, M., & Simpson, A. (2017). Towards a Principled Approach for Engineering Privacy by Design. In E. Schweighofer, H. Leitold, A. Mitrakas, & K. Rannenberg (Eds.), *Privacy Technologies and Policy* (Vol. 10518, pp. 161–177). https://doi.org/10.1007/978-3-319-67280-9_9

Antignac, T., Sands, D., & Schneider, G. (2016). Data Minimisation: A Language-Based Approach (Long Version). *ArXiv:1611.05642 [Cs]*. Retrieved from http://arxiv.org/abs/1611.05642

Arnold, M., Bellamy, R. K. E., Hind, M., Houde, S., Mehta, S., Mojsilovic, A., … Varshney, K. R. (2018). FactSheets: Increasing Trust in AI Services through Supplier’s Declarations of Conformity. *ArXiv:1808.07261 [Cs]*. Retrieved from http://arxiv.org/abs/1808.07261

Arnold, T, Kasenberg, D., & Scheutz, M. (2017). Value Alignment or Misalignment—What Will Keep Systems Accountable? *AAAI Workshops*.

Arnold, Thomas, & Scheutz, M. (2018). The “big red button” is too late: An alternative model for the ethical evaluation of AI systems. *Ethics and Information Technology*, *20*(1), 59–69. https://doi.org/10.1007/s10676-018-9447-7

Bach, S., Binder, A., Montavon, G., Klauschen, F., Müller, K.-R., & Samek, W. (2015). On Pixel-Wise Explanations for Non-Linear Classifier Decisions by Layer-Wise Relevance Propagation. *PLOS ONE*, *10*(7), e0130140. https://doi.org/10.1371/journal.pone.0130140

Bassily, R., Thakkar, O., & Thakurta, A. (2018). Model-Agnostic Private Learning via Stability. *ArXiv:1803.05101 [Cs]*. Retrieved from http://arxiv.org/abs/1803.05101

Bender, E. M., & Friedman, B. (2018). Data Statements for Natural Language Processing: Toward Mitigating System Bias and Enabling Better Science. *Transactions of the Association for Computational Linguistics*, *6*, 587–604. https://doi.org/10.1162/tacl_a_00041

Binns, R. (2018). Algorithmic Accountability and Public Reason. *Philosophy & Technology*, *31*(4), 543–556. https://doi.org/10.1007/s13347-017-0263-5

Binns, R. (n.d.). An Overview of the Auditing Framework for Artificial Intelligence and its core components. Retrieved from ICO website: https://ai-auditingframework.blogspot.com/2019/03/an-overview-of-auditing-framework-for_26.html

Bolukbasi, T., Chang, K., Zou, J., Saligrama, V., & Kalai. (2016). *Man is to Computer Programmer as Woman is to Homemaker? Debiasing Word Embeddings*. Presented at the NIPS.

Butnaru, C., Benrimoh, D., & Theodorou, A. (n.d.). Humans in AI. Retrieved from http://moralmachine.mit.edu/

Butterworth, M. (2018). The ICO and artificial intelligence: The role of fairness in the GDPR framework. *Computer Law & Security Review*, *34*(2), 257–268. https://doi.org/10.1016/j.clsr.2018.01.004

Calders, T., & Verwer, S. (2010). Three naive Bayes approaches for discrimination-free classification. *Data Mining and Knowledge Discovery*, *21*(2), 277–292. https://doi.org/10.1007/s10618-010-0190-x

Cavoukian, A., Taylor, S., & Abrams, M. E. (2010). Privacy by Design: Essential for organizational accountability and strong business practices. *Identity in the Information Society*, *3*(2), 405–413. https://doi.org/10.1007/s12394-010-0053-z

Chowdhury, R. (n.d.). Tackling the challenges of ethics in AI Fairness Tool. Retrieved from Accenture website: https://www.accenture.com/gb-en/blogs/blogs-cogx-tackling-challenge-ethics-ai

Citron, D., & Pasquale, F. (2014). The Scored Society: Due process for automated predictions. *Washington Law Review*, *89*(1), 1–33.

Datta, A., Sen, S., & Zick, Y. (2017). Algorithmic Transparency via Quantitative Input Influence. In T. Cerquitelli, D. Quercia, & F. Pasquale (Eds.), *Transparent Data Mining for Big and Small Data* (Vol. 32, pp. 71–94). https://doi.org/10.1007/978-3-319-54024-5_4

Dennis, L. A., Fisher, M., Lincoln, N. K., Lisitsa, A., & Veres, S. M. (2016). Practical verification of decision-making in agent-based autonomous systems. *Automated Software Engineering*, *23*(3), 305–359. https://doi.org/10.1007/s10515-014-0168-9

Diakopoulos, Nicholas. (2015). Algorithmic Accountability: Journalistic investigation of computational power structures. *Digital Journalism*, *3*(3), 398–415. https://doi.org/10.1080/21670811.2014.976411

Diakopoulos, Nicholas, Friedler, S., Arenas, M., Barocas, S., Howe, B., Jagadish, H., … Zevenbergen, B. (n.d.). Principles for Accountable Algorithms and a Social Impact Statement for Algorithms. Retrieved from FAT ML website: http://www.fatml.org/resources/principles-for-accountable-algorithms

Diakopoulos, Nick, Trielli, D., Yang, A., & Gao, A. (n.d.). Algorithm Tips—Resources and Leads for investigating algorithms in society. Retrieved from http://algorithmtips.org/about/

DotEveryone. (n.d.). The DotEveryone Consequence Scanning Agile Event. Retrieved from https://doteveryone.org.uk/project/consequence-scanning/

Ellpha. (n.d.). Retrieved from https://www.ellpha.com/

Enigma. (n.d.). Retrieved from https://enigma.co/

Epstein, Z., Payne, B. H., Shen, J. H., Hong, C. J., Felbo, B., Dubey, A., … Rahwan, I. (2018). TuringBox: An Experimental Platform for the Evaluation of AI Systems. *Proceedings of the Twenty-Seventh International Joint Conference on Artificial Intelligence*, 5826–5828. https://doi.org/10.24963/ijcai.2018/851

Equity Evaluation Corpus. (n.d.). Retrieved from https://saifmohammad.com/WebPages/Biases-SA.html

Ethics Net. (n.d.). Retrieved from https://www.ethicsnet.com/about

Feldman, M., Friedler, S., Moeller, J., Scheidegger, C., & Venkatasubramanian, S. (2014). Certifying and removing disparate impact. *ArXiv:1412.3756 [Cs, Stat]*. Retrieved from http://arxiv.org/abs/1412.3756

Fish, B., Kun, J., & Lelkes, Á. D. (2016). A Confidence-Based Approach for Balancing Fairness and Accuracy. *ArXiv:1601.05764 [Cs]*. Retrieved from http://arxiv.org/abs/1601.05764

Friedman, B., Hendry, D. G., & Borning, A. (2017). A Survey of Value Sensitive Design Methods. *Foundations and Trends® in Human–Computer Interaction*, *11*(2), 63–125. https://doi.org/10.1561/1100000015

Gebru, T., Morgenstern, J., Vecchione, B., Vaughan, J. W., Wallach, H., Daumeé III, H., & Crawford, K. (2018). Datasheets for Datasets. *ArXiv:1803.09010 [Cs]*. Retrieved from http://arxiv.org/abs/1803.09010

Glenn, J. (n.d.). Futures Wheel. Retrieved from Ethics Kit website: http://ethicskit.org/futures-wheel.html

Goldstein, A., Kapelner, A., Bleich, J., & Pitkin, E. (2013). Peeking Inside the Black Box: Visualizing Statistical Learning with Plots of Individual Conditional Expectation. *ArXiv:1309.6392 [Stat]*. Retrieved from http://arxiv.org/abs/1309.6392

Google. (n.d.). What if Tool. Retrieved from https://ai.googleblog.com/2018/09/the-what-if-tool-code-free-probing-of.html https://pair-code.github.io/what-if-tool/

Guidotti, R., Monreale, A., Ruggieri, S., Turini, F., Giannotti, F., & Pedreschi, D. (2018). A Survey of Methods for Explaining Black Box Models. *ACM Computing Surveys*, *51*(5), 1–42. https://doi.org/10.1145/3236009

Hall, P., & Gill, N. (n.d.). H2O.ai Machine Learning Interpretability Resources. Retrieved from https://github.com/h2oai/mli-resources/blob/master/notebooks/mono_xgboost.ipynb

Hazy. (n.d.). Retrieved from https://hazy.com/

Hesketh, P. (n.d.). Ethics Cards. Retrieved from Ethics Kit website: http://ethicskit.org/ethics-cards.html

Holland, S., Hosny, A., Newman, S., Joseph, J., & Chmielinski, K. (2018). The Dataset Nutrition Label: A Framework To Drive Higher Data Quality Standards. *ArXiv:1805.03677 [Cs]*. Retrieved from http://arxiv.org/abs/1805.03677

ICO. (n.d.-a). *Anonymisation: Managing data protection riskcode of practice*.

ICO. (n.d.-b). Guide to the General Data Protection Regulation (GDPR). Retrieved from https://ico.org.uk/for-organisations/guide-to-data-protection/guide-to-the-general-data-protection-regulation-gdpr/

Ideo.org. (n.d.). The field guide to human-centred design. Retrieved from http://www.designkit.org/resources/1

IEEE. (n.d.). *Artificial Intelligence and Ethics in Design Course Program*. Retrieved from https://innovationatwork.ieee.org/courses/artificial-intelligence-and-ethics-in-design/

Involve, & DeepMind. (n.d.). *How to stimulate effective public engagement on the ethics of Artificial Intelligence*. Retrieved from https://www.involve.org.uk/sites/default/files/field/attachemnt/How%20to%20stimulate%20effective%20public%20debate%20on%20the%20ethics%20of%20artificial%20intelligence%20.pdf

Johansson, F. D., Shalit, U., & Sontag, D. (2016). Learning Representations for Counterfactual Inference. *ArXiv:1605.03661 [Cs, Stat]*. Retrieved from http://arxiv.org/abs/1605.03661

Joshi, C., Kaloskampis, I., & Nolan, L. (2019). Generative Adversarial Networks (GANs) for synthetic dataset generation with binary classes. Retrieved from https://datasciencecampus.ons.gov.uk/projects/generative-adversarial-networks-gans-for-synthetic-dataset-generation-with-binary-classes/

Kleinberg, J., Lakkaraju, H., Leskovec, J., Ludwig, J., & Mullainathan, S. (2017). Human Decisions and Machine Predictions*. *The Quarterly Journal of Economics*. https://doi.org/10.1093/qje/qjx032

Kolter, Z., & Madry, A. (n.d.). Materials for tutorial Adversarial Robustness: Theory and Practice. Retrieved from https://adversarial-ml-tutorial.org/

Kroll, J. A. (2018). The fallacy of inscrutability. *Philosophical Transactions of the Royal Society A: Mathematical, Physical and Engineering Sciences*, *376*(2133), 20180084. https://doi.org/10.1098/rsta.2018.0084

Kroll, J. A., Huey, J., Barocas, S., Felten, E., Reidenberg, J., Robinson, D., & Yu, H. (2017). Accountable Algorithms. *University of Pennyslvania Law Review*, *165*.

Kusner, M. J., Loftus, J. R., Russell, C., & Silva, R. (2017). Counterfactual Fairness. *ArXiv:1703.06856 [Cs, Stat]*. Retrieved from http://arxiv.org/abs/1703.06856

Lakkaraju, H., Kleinberg, J., Leskovec, J., Ludwig, J., & Mullainathan, S. (2017). The Selective Labels Problem: Evaluating Algorithmic Predictions in the Presence of Unobservables. *Proceedings of the 23rd ACM SIGKDD International Conference on Knowledge Discovery and Data Mining - KDD ’17*, 275–284. https://doi.org/10.1145/3097983.3098066

Lepri, B., Oliver, N., Letouzé, E., Pentland, A., & Vinck, P. (2018). Fair, Transparent, and Accountable Algorithmic Decision-making Processes: The Premise, the Proposed Solutions, and the Open Challenges. *Philosophy & Technology*, *31*(4), 611–627. https://doi.org/10.1007/s13347-017-0279-x

Li, O., Liu, H., Chen, C., & Rudin, C. (2017). Deep Learning for Case-Based Reasoning through Prototypes: A Neural Network that Explains Its Predictions. *ArXiv:1710.04806 [Cs, Stat]*. Retrieved from http://arxiv.org/abs/1710.04806

Lundberg, S., & Lee, S.-I. (2017). A Unified Approach to Interpreting Model Predictions. *ArXiv:1705.07874 [Cs, Stat]*. Retrieved from http://arxiv.org/abs/1705.07874

Madras, D., Creager, E., Pitassi, T., & Zemel, R. (2018). Learning Adversarially Fair and Transferable Representations. *ArXiv:1802.06309 [Cs, Stat]*. Retrieved from http://arxiv.org/abs/1802.06309

Makri, E.-L., & Lambrinoudakis, C. (2015). Privacy Principles: Towards a Common Privacy Audit Methodology. In S. Fischer-Hübner, C. Lambrinoudakis, & J. López (Eds.), *Trust, Privacy and Security in Digital Business* (Vol. 9264, pp. 219–234). https://doi.org/10.1007/978-3-319-22906-5_17

Microsoft. (n.d.). InterpretML - Alpha Release. Retrieved from GitHub website: https://github.com/Microsoft/interpret

MIT. (n.d.). Moral Machines. Retrieved from http://moralmachine.mit.edu/

Mitchell, M., Wu, S., Zaldivar, A., Barnes, P., Vasserman, L., Hutchinson, B., … Gebru, T. (2019). Model Cards for Model Reporting. *Proceedings of the Conference on Fairness, Accountability, and Transparency - FAT* ’19*, 220–229. https://doi.org/10.1145/3287560.3287596

New Economy Impact Model. (n.d.). Retrieved from The Federation website: http://ethicskit.org/downloads/economy-impact-model.pdf

Nicolae, M.-I., Sinn, M., Tran, M. N., Rawat, A., Wistuba, M., Zantedeschi, V., … Edwards, B. (2018). Adversarial Robustness Toolbox v0.4.0. *ArXiv:1807.01069 [Cs, Stat]*. Retrieved from http://arxiv.org/abs/1807.01069

ODI. (n.d.). Data Ethics Canvas User Guide. Retrieved from https://docs.google.com/document/d/1MkvoAP86CwimbBD0dxySVCO0zeVOput_bu1A6kHV73M/edit

Oetzel, M. C., & Spiekermann, S. (2014). A systematic methodology for privacy impact assessments: A design science approach. *European Journal of Information Systems*, *23*(2), 126–150. https://doi.org/10.1057/ejis.2013.18

ONS. (n.d.). The ONS Methodology working paper on Synthetic Data. Retrieved from https://www.ons.gov.uk/methodology/methodologicalpublications/generalmethodology/onsworkingpaperseries/onsmethodologyworkingpaperseriesnumber16syntheticdatapilot

OpenMined. (n.d.). Retrieved from https://www.openmined.org/

Orcutt, M. (2017). Personal AI Privacy Watchdog Could Help You Regain Control of Your Data. *MIT Technology Review*. Retrieved from https://www.technologyreview.com/s/607830/personal-ai-privacy-watchdog-could-help-you-regain-control-of-your-data/

Overdorf, R., Kulynych, B., Balsa, E., Troncoso, C., & Gürses, S. (2018). Questioning the assumptions behind fairness solutions. *ArXiv:1811.11293 [Cs]*. Retrieved from http://arxiv.org/abs/1811.11293

Oxborough, C., Cameron, E., Rao, A., Birchall, A., Townsend, A., & Westermann, C. (n.d.). *Explainable AI: Driving Business Value through Greater Understanding*. Retrieved from PWC website: https://www.pwc.co.uk/audit-assurance/assets/explainable-ai.pdf

Papernot, N., Song, S., Mironov, I., Raghunathan, A., Talwar, K., & Erlingsson, Ú. (2018). Scalable Private Learning with PATE. *ArXiv:1802.08908 [Cs, Stat]*. Retrieved from http://arxiv.org/abs/1802.08908

Peters, D., & Calvo, R. A. (2019, May 2). Beyond principles: A process for responsible tech. Retrieved from Medium website: https://medium.com/ethics-of-digital-experience/beyond-principles-a-process-for-responsible-tech-aefc921f7317

Peters, D., Calvo, R. A., & Ryan, R. M. (2018). Designing for Motivation, Engagement and Wellbeing in Digital Experience. *Frontiers in Psychology*, *9*, 797. https://doi.org/10.3389/fpsyg.2018.00797

Pineau, J. (2019). The Machine Learning Reproducibility Checklist. Retrieved from https://www.cs.mcgill.ca/~jpineau/ReproducibilityChecklist.pdf

Reisman, D., Schultz, J., Crawford, K., & Whittaker, M. (2018). *Algorithmic Impact Assessments: A Practical Framework for Public Agency Accountability*. Retrieved from AINow website: https://ainowinstitute.org/aiareport2018.pdf

Responsible AI Licenses. (n.d.). Retrieved from https://www.licenses.ai/about

Ribeiro, M. T., Singh, S., & Guestrin, C. (2016a). “Why Should I Trust You?”: Explaining the Predictions of Any Classifier. *ArXiv:1602.04938 [Cs, Stat]*. Retrieved from http://arxiv.org/abs/1602.04938

Ribeiro, Singh, S., & Guestrin, C. (2016b, August 12). Local Interpretable Model-Agnostic Explanations (LIME): An Introduction A technique to explain the predictions of any machine learning classifier. Retrieved from https://www.oreilly.com/learning/introduction-to-local-interpretable-model-agnostic-explanations-lime

Royal Society, & British Academy. (n.d.). *Data Management and Use: Governance in the 21st Century*. Retrieved from https://royalsociety.org/~/media/policy/projects/data-governance/data-management-governance.pdf

Russell, C., Kusner, M. J., Loftus, J., & Silva, R. (2017). When Worlds Collide: Integrating Different Counterfactual Assumptions in Fairness. In I. Guyon, U. V. Luxburg, S. Bengio, H. Wallach, R. Fergus, S. Vishwanathan, & R. Garnett (Eds.), *Advances in Neural Information Processing Systems 30* (pp. 6414–6423). Retrieved from http://papers.nips.cc/paper/7220-when-worlds-collide-integrating-different-counterfactual-assumptions-in-fairness.pdf

Ryffel, T., Trask, A., Dahl, M., Wagner, B., Mancuso, J., Rueckert, D., & Passerat-Palmbach, J. (2018). A generic framework for privacy preserving deep learning. *ArXiv:1811.04017 [Cs, Stat]*. Retrieved from http://arxiv.org/abs/1811.04017

Saleiro, P., Kuester, B., Stevens, A., Anisfeld, A., Hinkson, L., London, J., & Ghani, R. (2018). Aequitas: A Bias and Fairness Audit Toolkit. *ArXiv:1811.05577 [Cs]*. Retrieved from http://arxiv.org/abs/1811.05577

Sampson, O., & Chapman, M. (2019, May 8). AI Needs an Ethical Compass. This Tool Can Help. Retrieved from Ideo website: https://www.ideo.com/blog/ai-needs-an-ethical-compass-this-tool-can-help

Sandvig, C., Hamilton, K., Karahalios, K., & Langbort, C. (2014). *Auditing Algorithms: Research Methods for Detecting Discrimination on Internet Platforms*. Presented at the Data and Discrimination: Converting Critical Concerns into Productive Inquiry” a preconference at the 64th Annual Meeting of the International Communication Association Seattle, WA, USA.

Seldon.io. (n.d.). Alibi. Retrieved from GitHub website: https://github.com/SeldonIO/alibi

Shrikumar, A., Greenside, P., & Kundaje, A. (2017). Learning Important Features Through Propagating Activation Differences. *ArXiv:1704.02685 [Cs]*. Retrieved from http://arxiv.org/abs/1704.02685

Simonyan, K., Vedaldi, A., & Zisserman, A. (2013). Deep Inside Convolutional Networks: Visualising Image Classification Models and Saliency Maps. *ArXiv:1312.6034 [Cs]*. Retrieved from http://arxiv.org/abs/1312.6034

Sokol, K., & Flach, P. (2018). Glass-Box: Explaining AI Decisions With Counterfactual Statements Through Conversation With a Voice-enabled Virtual Assistant. *Proceedings of the Twenty-Seventh International Joint Conference on Artificial Intelligence*, 5868–5870. https://doi.org/10.24963/ijcai.2018/865

Stahl, B. C., & Wright, D. (2018). Ethics and Privacy in AI and Big Data: Implementing Responsible Research and Innovation. *IEEE Security & Privacy*, *16*(3), 26–33. https://doi.org/10.1109/MSP.2018.2701164

Suphakul, T., & Senivongse, T. (2017). Development of privacy design patterns based on privacy principles and UML. *2017 18th IEEE/ACIS International Conference on Software Engineering, Artificial Intelligence, Networking and Parallel/Distributed Computing (SNPD)*, 369–375. https://doi.org/10.1109/SNPD.2017.8022748

TensorFlow Privacy. (n.d.). Retrieved from https://github.com/tensorflow/privacy

The Turing Way. (n.d.). Retrieved from https://github.com/alan-turing-institute/the-turing-way

van de Poel, I. (2016). An Ethical Framework for Evaluating Experimental Technology. *Science and Engineering Ethics*, *22*(3), 667–686. https://doi.org/10.1007/s11948-015-9724-3

Varshney, K. R. (2018). Introducing AI Fairness 360. Retrieved from IBM website: https://www.ibm.com/blogs/research/2018/09/ai-fairness-360/ https://aif360.mybluemix.net/

Wachter, S., & Mittelstadt, B. (n.d.). A Right to Reasonable Inferences: Re-Thinking Data Protection Law in the Age of Big Data and AI (September 13,2018). *Columbia Business Law Review, Forthcoming*. Retrieved from https://ssrn.com/abstract=3248829

Wachter, S., Mittelstadt, B., & Russell, C. (2017). Counterfactual Explanations without Opening the Black Box: Automated Decisions and the GDPR. *ArXiv:1711.00399 [Cs]*. Retrieved from http://arxiv.org/abs/1711.00399

Wellcome Data Labs. (n.d.). A new method for ethical data science. Retrieved from https://medium.com/wellcome-data-labs/a-new-method-for-ethical-data-science-edb59e400ae9

Wexler, J. (2018). The What-If Tool: Code-Free Probing of Machine. Retrieved from https://ai.googleblog.com/2018/09/the-what-if-tool-code-free-probing-of.html. https://pair-code.github.io/what-if-tool/

Wilson, C. (2018). Auditing Algorithms @ Northeastern. Retrieved from http://personalization.ccs.neu.edu/

XAI Library. (n.d.). Retrieved from https://github.com/EthicalML/awesome-machine-learning-operations

Zafar, M. B., Valera, I., Rodriguez, M. G., & Gummadi, K. P. (2015). Fairness Constraints: Mechanisms for Fair Classification. *ArXiv:1507.05259 [Cs, Stat]*. Retrieved from http://arxiv.org/abs/1507.05259

Zhang, Q., & Zhu, S. (2018). Visual interpretability for deep learning: A survey. *Frontiers of Information Technology & Electronic Engineering*, *19*(1), 27–39. https://doi.org/10.1631/FITEE.1700808

Zhao, W.-W. (2018). Improving Social Responsibility of Artificial Intelligence by Using ISO 26000. *IOP Conference Series: Materials Science and Engineering*, *428*, 012049. https://doi.org/10.1088/1757-899X/428/1/012049

Zook, M., Barocas, S., boyd, danah, Crawford, K., Keller, E., Gangadharan, S. P., … Pasquale, F. (2017). Ten simple rules for responsible big data research. *PLOS Computational Biology*, *13*(3), e1005399. https://doi.org/10.1371/journal.pcbi.1005399

Zyskind, G., Nathan, O., & Pentland, A. (2015). Enigma: Decentralized Computation Platform with Guaranteed Privacy. *ArXiv:1506.03471 [Cs]*. Retrieved from http://arxiv.org/abs/1506.03471
